# Supplementary material for: Self-powered and speed-adjustable sensor for abyssal ocean current measurements based on triboelectric nanogenerators
Source: Nat Commun. 2024 Jul 20;15:6133. doi: 10.1038/s41467-024-50581-w (PMC11271462; doi:10.1038/s41467-024-50581-w)
Supplement: Supplementary file 1 — Supplementary Information [file 41467_2024_50581_MOESM1_ESM.pdf]

Supplementary Information

Self-powered and speed-adjustable sensor for abyssal ocean current measurements based on triboelectric nanogenerators

Yuan Chao Pan[1,2], Zhuhang Dai[1], Haoxiang Ma[1], Jinrong Zheng[1], Jing Leng[1], Chao Xie[1], Yapeng Yuan[1], Wencai Yang[1], Yaxiaer Yalikun[3], Xuemei Song[2], Chang Bao Han[2,*], Chenjing Shang[4,1,*], and Yang Yang[1,5,*]

[1]Institute of Deep-Sea Science and Engineering, Chinese Academy of Sciences, 572000 Sanya, China

[2]The Key Laboratory of Advanced Functional Materials, Ministry of Education of China, Faculty of Materials and Manufacturing, Beijing University of Technology, Beijing 100124, People's Republic of China

[3]Division of Materials Science, Nara Institute of Science and Technology, 8916–5 Takayama-cho, Ikoma, Nara 630–0192, Japan

[4]Shenzhen Key Laboratory of Marine Bioresource and Eco-environmental Science, College of Life Science and Oceanography, Shenzhen University, Shenzhen 518060, China

[5]Lead Contact

*Correspondence: cbhan@bjut.edu.cn (C.B.H.); cjshang@szu.edu.cn (C.S.); yangyang@idsse.ac.cn (Y.Y.)

Content

Supplementary Figures

Supplementary Fig. 1. Effect of fur length on the contact area.

Supplementary Fig. 2. Long-term output (induced charge) stability performance of the DS-TENG.

Supplementary Fig. 3. SEM image of rabbit fur after running for more than 30 days.

Supplementary Fig. 4. Transmission curve at different transmission clearances. (a) 0 mm, (b) 12.5 mm, (c) 15.5 mm, (d) 18.5 mm, (e) 21.5 mm, (f) 24.5 mm.

Supplementary Fig. 5. Diagram of influence of axial force of magnetic coupling on transmission resistance.

Supplementary Fig. 6. Output performance of the DS-TENG. (a) Open-circuit voltage, (b) short-circuit current, and (c) transferred charge of the DS-TENG at 1200~2000 rpm.

Supplementary Fig. 7. Liner relationship between the rotating-cup speed and relative velocity of the DS-TENG. (a) State 1; (b) State 2. Error bars represent standard deviation over three independent measurements.

Supplementary Fig. 8. Photograph of the DS-TENG mounted on the ROV.

Supplementary Fig. 9. The circuit diagram of the DS-TENG.

Supplementary Fig. 10. Original output signal waveform of the DS-TENG when the ROV is stably traveling at 0.30 m/s.

Supplementary Fig. 11. The detailed design parameters of the rotating cups of DS-TENG.

Supplementary Fig. 12. Schematic of the test device.

Supplementary Movies

Supplementary Movie 1. DS-TENG maintaining stable output performance at high ship speeds during sea trials.

Supplementary Movie 2. DS-TENG demonstrating excellent sensitivity at low ship speeds during sea trials.

Supplementary Movie 3. DS-TENG descending into the sea along with the ROV.

Supplementary Movie 4. The DS-TENG resurfacing from the sea along with the ROV.

Supplementary Movie 5. DS-TENG operating stably at a depth of 4531 m while the ROV travels at a speed of 0.35 m/s.

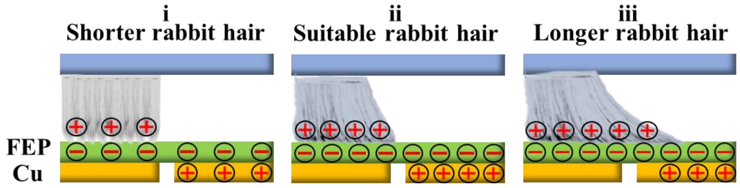

Supplementary Fig. 1. Effect of fur length on the contact area.

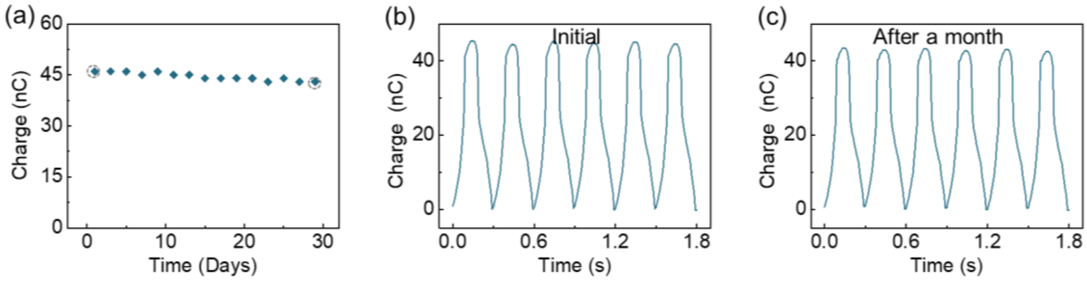

Supplementary Fig. 2. Long-term output (induced charge) stability performance of the DS-TENG.

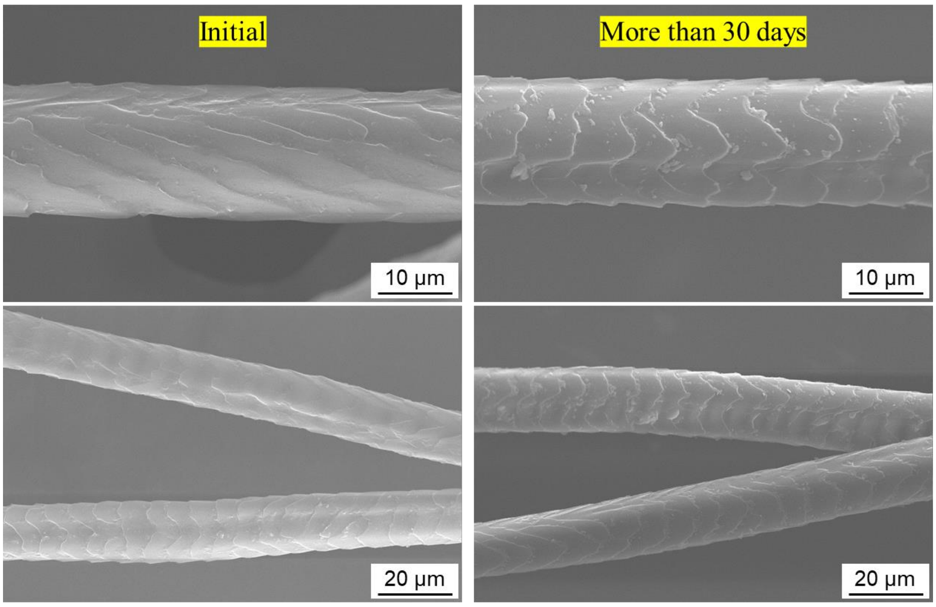

Supplementary Fig. 3. SEM image of rabbit fur after running for more than 30 days.

The output performance of the TENG is affected by external environmental factors and TENG's inherent factors. Environmental factors primarily comprise temperature, humidity, radiation, and electromagnetic interference. This manuscript does not consider external environmental factors (e.g. humidity, pressure, corrosiveness) because the TENG is isolated in a sealed tank.

TENG's inherent factors are described as follows.

(1) Material and wear factors: First, the two friction materials (FEP and fur) have good chemical stability such that they will generally last many years. Second, the materials have a good abrasive resistance. In principle, during the long-term friction process, the two materials rubbing against each other leave material debris on one another, which may reduce the surface charge

density of the tribomaterials and thereby affect the TENG output. Given the rabbit fur and FEP used in this study have high wear resistance, and it is a slight contact friction between them, therefore, material and wear factors do not greatly affect the material lifetimes.

(2) Structural factors: The amount of triboelectric charge is related to the degree of contact between the two friction materials. The rabbit fur used in this study makes good contact with the FEP in the initial stage. With an increase in the running time, the contact force between the rabbit fur and FEP decreases because of the poor elastic restoring force, which reduces the area of contact and thus attenuates the output charge. Theoretically, the lifetime of the TENG might be over 10 years if the performance decay due to contact is avoided. Therefore, to increase the lifetime of the DS-TENG, the rabbit fur structure can be further optimized using following methods: (1) by surface treatment of the rabbit fur, or by further reducing the fur length to increase the fur's elasticity for long-term stable contact with the FEP; (2) by replacing the rabbit fur with other polymer brush structures with better elasticity to ensure a stable contact in the long-term.

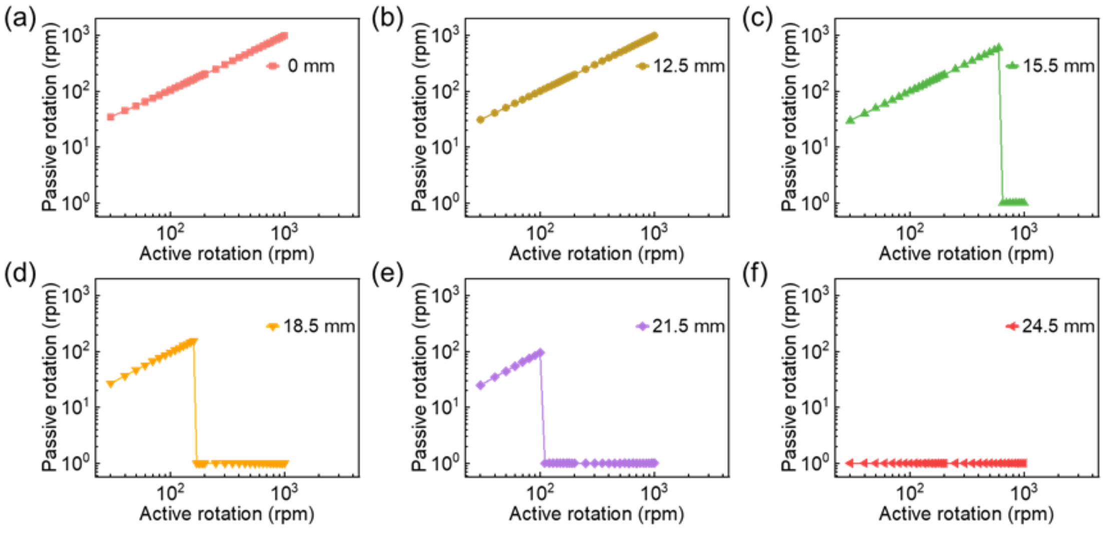

Supplementary Fig. 4. Transmission curve at different transmission clearances. (a) 0 mm, (b) 12.5 mm, (c) 15.5 mm, (d) 18.5 mm, (e) 21.5 mm, (f) 24.5 mm.

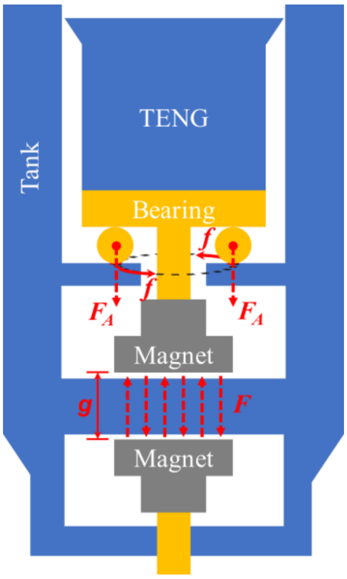

Supplementary Fig. 5. Diagram of influence of axial force of magnetic coupling on transmission resistance.

According to the transmission principle of the magnetic coupling, the two magnets of the magnetic coupling generate an axial force that attracts each magnet to the other in addition to transmitting torque. As shown in Fig. R5, the axial force increases the frictional resistance of the internal structure. In addition, as the transmission clearance decreases, the axial force gradually increases, resulting in greater internal frictional resistance and therefore worse start-up performance at low flow velocity. Therefore, at low flow velocity, we use a large clearance to reduce frictional resistance and thus achieve better start-up. Moreover, according to the acceleration formula $V_t = V_0 + at$ and $F = ma$, we have $F = mV_t/t$ when $V_0 = 0$ (where V_t is the final velocity, V_0 is the initial velocity, a is the acceleration, m is the mass of the object, and t is the time required to reach V_t). This indicates that the time required for the cup to reach the rotating speed (V_t) corresponding to a certain flow velocity is inversely proportional to the thrust of the flow (F). Therefore, owing to the small F at low flow velocity, a long response time is required. Indeed, a large transmission clearance corresponds to an extended response time.

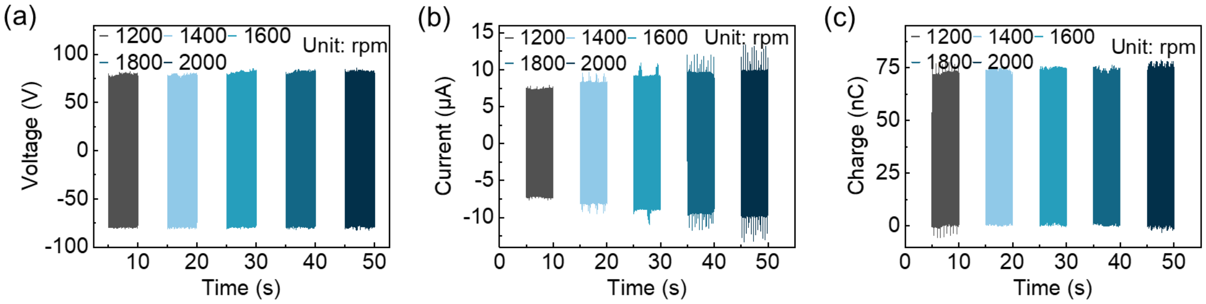

Supplementary Fig. 6. Output performance of the DS-TENG.

(A) Open-circuit voltage, (B) short-circuit current, and (C) transferred charge of the DS-TENG at 1200~2000 rpm.

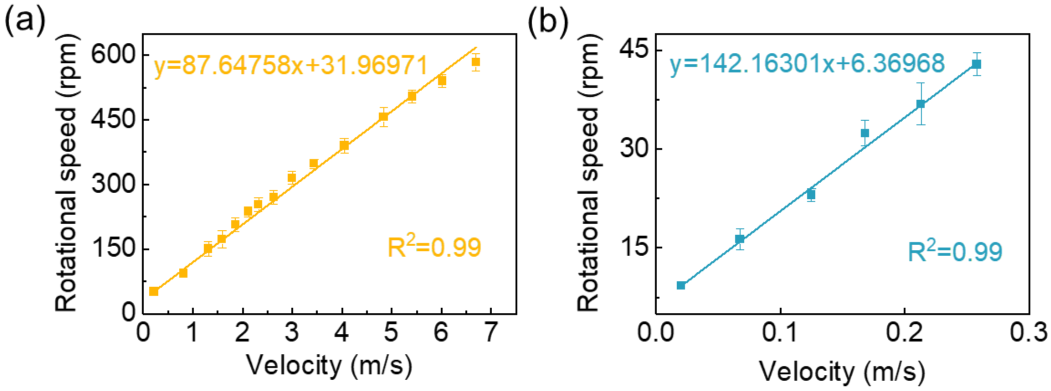

Supplementary Fig. 7. Liner relationship between the rotating-cup speed and relative velocity of the DS-TENG.

(a) State 1; (b) State 2. Error bars represent standard deviation over three independent measurements.

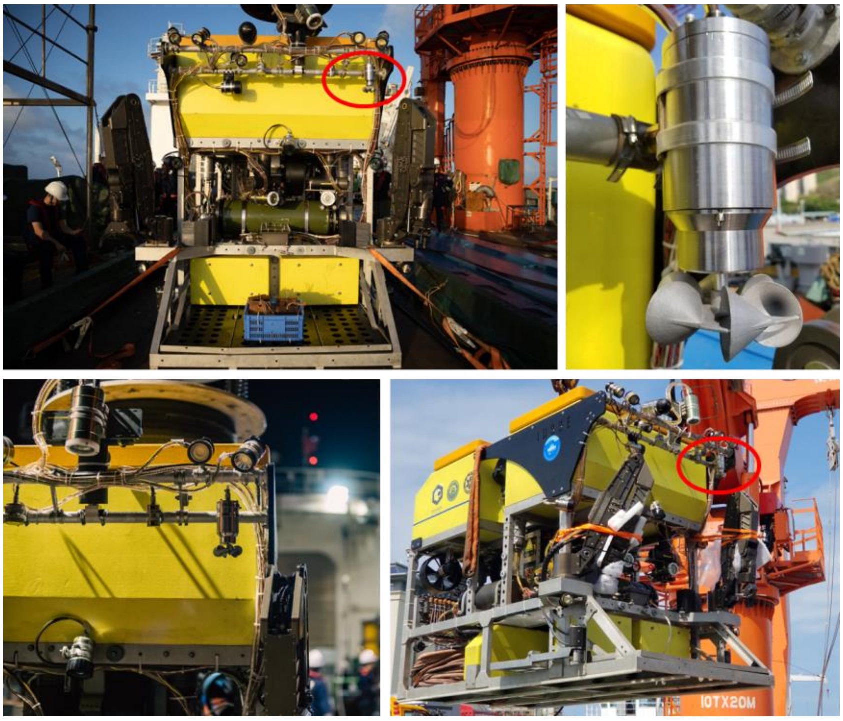

Supplementary Fig. 8. Photograph of the DS-TENG mounted on the ROV.

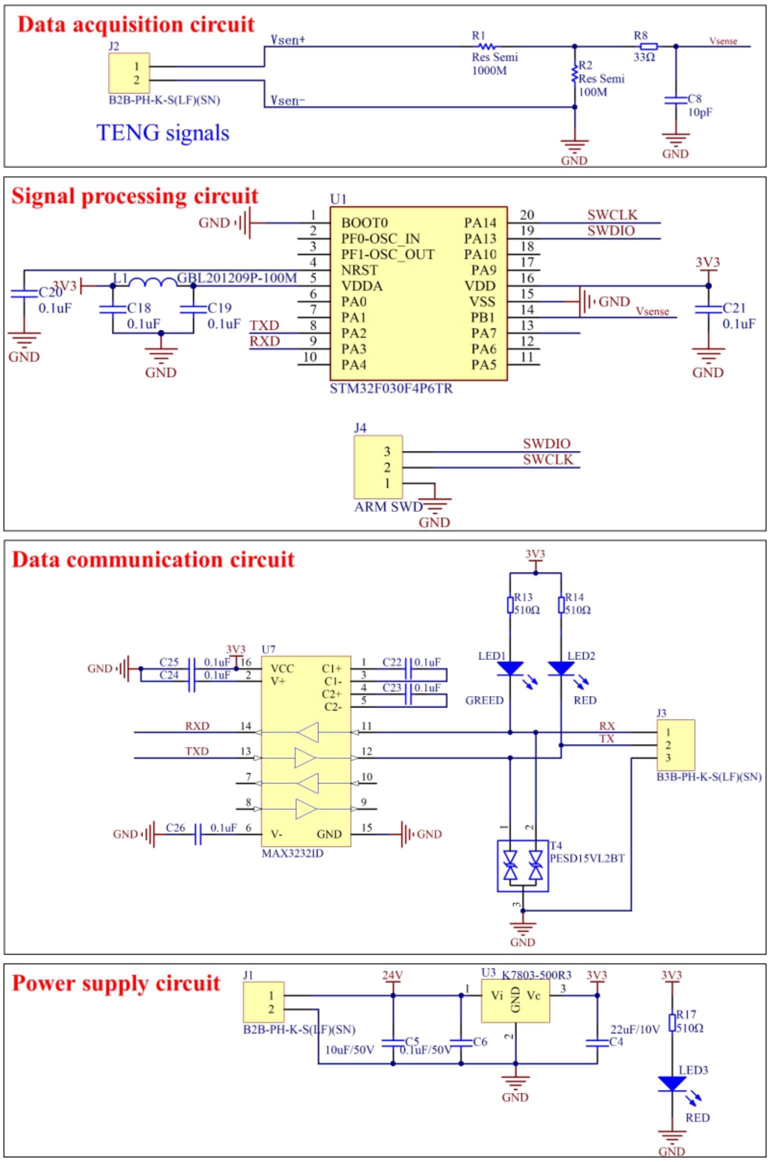

Supplementary Fig. 9. The circuit diagram of the DS-TENG.

The circuit board includes an acquisition circuit, processing circuit, and communication circuit. The function of the acquisition circuit is to acquire the high-resistance and high-voltage signals of the TENG. First, the acquisition circuit divides and filters the TENG signals. Then, the microcontroller in the processing circuit performs analog-to-digital conversion and data encoding of analog signals through an analog-to-digital converter. Finally, the communications circuit further transmits the encapsulated data to the ground platform via the ROV platform.

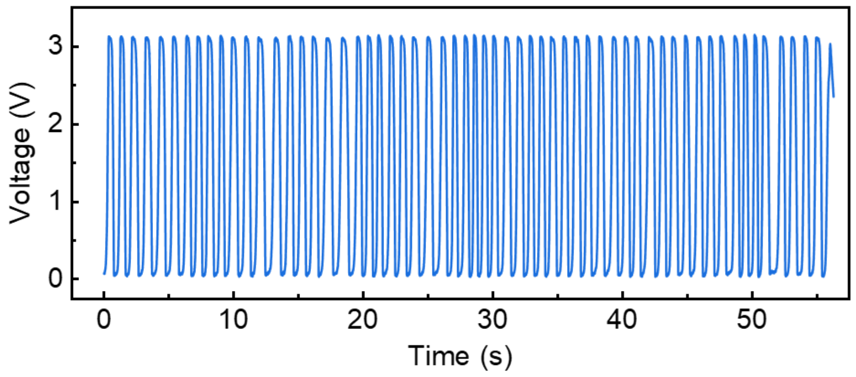

Supplementary Fig. 10. Original output signal waveform of the DS-TENG when the ROV is stably traveling at 0.30 m/s.

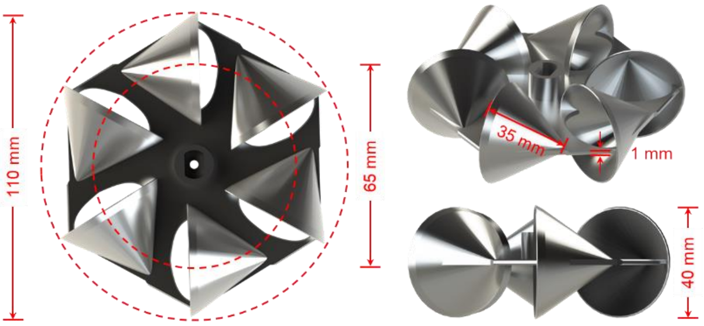

Supplementary Fig. 11. The detailed design parameters of the rotating cups of DS-TENG.

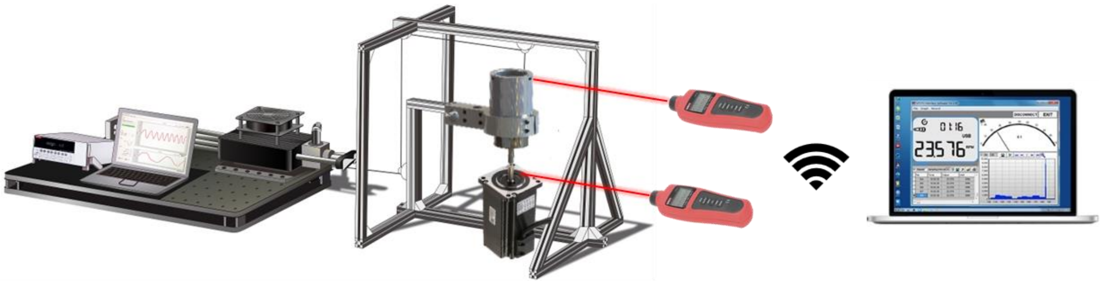

Supplementary Fig. 12. Schematic of the test device.
