# Supplementary material for: Self-powered and speed-adjustable sensor for abyssal ocean current measurements based on triboelectric nanogenerators
Source: Nat Commun. 2024 Jul 20;15:6133. doi: 10.1038/s41467-024-50581-w (PMC11271462; doi:10.1038/s41467-024-50581-w)
Supplement: Supplementary file 3 — Description of Additional Supplementary Files [file 41467_2024_50581_MOESM3_ESM.pdf]

Description of Additional Supplementary Files

Supplementary Movie 1. DS-TENG maintaining stable output performance at high ship speeds during sea trials.

Supplementary Movie 2. DS-TENG demonstrating excellent sensitivity at low ship speeds during sea trials.

Supplementary Movie 3. DS-TENG descending into the sea along with the ROV.

Supplementary Movie 4. The DS-TENG resurfacing from the sea along with the ROV.

Supplementary Movie 5. DS-TENG operating stably at a depth of 4531 m while the ROV travels at a speed of 0.35 m/s.
